# Except for my commute, everything is the same: the shared lived experience of health sciences libraries during the COVID-19 pandemic

**DOI:** 10.5195/jmla.2022.1475

**Published:** 2022-10-01

**Authors:** Bart Ragon, Elizabeth C. Whipple, Melissa L. Rethlefsen

**Affiliations:** 1 bart@virginia.edu, Director, Claude Moore Health Sciences Library, University of Virginia, Charlottesville, VA.; 2 ewhipple@iu.edu, Assistant Director for Research & Translational Sciences, Ruth Lilly Medical Library, Indiana University School of Medicine, Indianapolis, IN.; 3 mlrethlefsen@gmail.com, Executive Director, Health Sciences Library & Informatics Center, University of New Mexico, Albuquerque, NM.

**Keywords:** Libraries, medical, health sciences librarians, COVID-19

## Abstract

**Objective:**

To understand the experience of academic health sciences libraries during the pandemic using a phenomenological approach.

**Methods:**

This study used a multisite, mixed-method approach to capture the direct experience of academic health sciences libraries as they evolved during the COVID-19 pandemic. Phase one of the study involved administering a qualitative survey to capture to capture current evolutions of programs and services. The survey for phases two (August 2020) and three (February 2021) contained eight questions asking participants to share updates on their evolution and experiences.

**Results:**

Qualitative data were analyzed using open coding techniques to ensure emergent themes were allowed to surface. Additional post-hoc sentiment analysis ascertained the frequency of positive and negative words in each data set. Of the 193 possible AAHSL libraries, 45 (23.3%) responded to the April 2020 survey, 26 to the August 2020 survey, and 16 to the February 2021 survey. Libraries represented 23 states and the District of Columbia. The majority of libraries closed in March 2020. The ease of transferring library services to a remote environment varied by type of service. For the quantitative analysis, ten distinct areas were analyzed using text coded as “Staff” as a lens for understanding the connection between codes.

**Conclusion:**

Innovations by libraries during the early stages of the pandemic are having a long-term impact on library culture and the delivery of services. Even as libraries returned to in-person services, elements of telecommuting, using online conferencing software, safety precautions, and monitoring of staff well-being persisted.

## INTRODUCTION

The global COVID-19 pandemic has wrought unprecedented changes to our communities and personal and work lives. In March 2020, the World Health Organization (WHO) declared COVID-19 as a pandemic and recommended social distancing as a means to curb the spread; this pandemic caused many North American institutions of higher education to rapidly transition to remote learning and services, shutter nonessential buildings, and restrict campus access [1, 2]. For campuses with health systems and hospitals, there was a desperate rush to learn about the new coronavirus and how to detect and treat COVID-19, even as many campus research labs were forced to shut down [3]. Shortages in personal protective equipment and other vital resources forced health care systems and hospitals to alter their supply chains and be creative about obtaining scarce materials such as cleaning supplies and hand sanitizer [4]. The economics of the pandemic quickly became apparent as hospitals implemented crisis standards of care, universities returned housing and dining payments to students, and costs for newly needed supplies skyrocketed. Institutions grappled with research shutdowns, high demand for video conferencing tools, switching to fully online learning environments, and nearly hourly changes in the understanding of the coronavirus and COVID-19.

By mid-March, it was clear academic health sciences libraries were in similar upheaval as they scrambled to figure out how to work within the confines of the pandemic. Listservs abounded with messages of libraries' physical closures as COVID-19 swept through the continent. In a matter of days or sometimes hours, libraries had to make hard decisions regarding how—and what—to move to remote environments. As April 2020 neared, it was increasingly clear the short shutdown many anticipated was too optimistic; the COVID-19 pandemic would last longer than anticipated or hoped. Indeed, by the end of April 2020, Johns Hopkins reported the United States surpassed one million confirmed cases [5]. Even by the end of March 2020, it was evident the pandemic would have substantial, ongoing impact on health sciences libraries and their services. The phenomenological approach used in this study sought to understand the experience of academic health sciences libraries during the pandemic by gathering data about the state of libraries at key points of the pandemic. The use of survey instruments allowed the study to capture a broad range of perspectives from multiple organizations as changes occurred.

## METHODS

This study used a multisite, mixed-method approach to capture the direct experience of academic health sciences libraries as they evolved during the COVID-19 pandemic. The study was approved by University of Virginia's IRB #3639. An email soliciting participation and describing the research project, its purpose, and participants' rights was sent to the Association of Academic Health Sciences Libraries (AAHSL) listserv. AAHSL was chosen as its members broadly represent leadership across academic health sciences libraries in the United States and Canada. This study gathered the perspectives of administrative leaders at academic health sciences libraries, who were tasked with leading and decision making when responding to the crisis.

Participants of the study agreed to respond to multiple surveys to assist in understanding the impact of the pandemic on libraries over time. Data were collected in April 2020, August 2020, and February 2021. Phase one of the study involved a qualitative survey capturing current evolutions of programs and services. The initial April 2020 survey asked 20 questions and invited respondents to participate in the next two surveys (see Data Availability Statement). The surveys for phases two and three contained eight questions asking participants to share updates on their evolution and experiences (see Supplemental Appendix A). The August 2020 and February 2021 surveys were identical and developed based on answers to the April 2020 survey, emphasizing the themes and topics seemingly prominent within the first survey's data.

All survey responses were collected in Qualtrics. The quantitative data from all three surveys were analyzed using basic descriptive statistics in Microsoft Excel. The qualitative data (free text responses) were coded using Dedoose, an online platform for analyzing qualitative and mixed-methods research. The open-ended survey responses were coded by two researchers, who used open coding techniques to ensure emergent themes were allowed to surface. Codes and definitions were refined and clarified throughout the process, and previously coded data were reviewed to ensure quality and consistency. Independent validation of a randomized sample of the data by a third researcher helped ensure inter-rater reliability.

Descriptive statistics of code occurrence and co-occurrence assisted in illuminating important themes and trends for libraries (see Table 4). Ten distinct areas were analyzed using text coded as “Staff” as a lens for understanding the connection between codes. Data were clustered into the following areas: remote library services, library facilities, and planning to reopen; internal communication and wellness and well-being; lessons learned, skills needed, and telecommuting; budget reduction and uncertainty; issues related to equity.

**Table 1:** April 2020 Library Status by Region.

	East n (%)	Midwest n (%)	South n (%)	West n (%)	Total n (%)
Library is completely closed	9 (75.0)	5 (62.5)	3 (37.5)	4 (44.4)	21 (56.8)
Library is closed, but a 24 hour space is open	3 (25.0)	0 (0.0)	1 (12.5)	2 (22.2)	6 (16.2)
Library is closed, but one or more staff are regularly working in the facility	0 (0.0)	3 (37.5)	5 (62.5)	5 (55.6)	13 (35.1)
Library is open with reduced staffing	0 (0.0)	0 (0.0)	0 (0.0)	1 (11.1)	1 (2.7)
Total Libraries responding	12 (32.4)	8 (21.6)	8 (21.6)	9 (24.3)	37 (100.0)

**Table 2:** Impact of COVID-19 on library operations, finances, and staffing by region, comparing August 2020 and February 2021 responses.

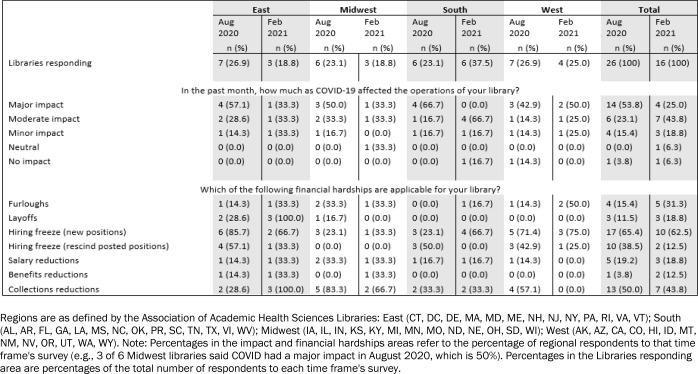

Regions are as defined by the Association of Academic Health Sciences Libraries: East (CT, DC, DE, MA, MD, ME, NH, NJ, NY, PA, RI, VA, VT); South (AL, AR, FL, GA, LA, MS, NC, OK, PR, SC, TN, TX, VI, WV); Midwest (IA, IL, IN, KS, KY, MI, MN, MO, ND, NE, OH, SD, WI); West (AK, AZ, CA, CO, HI, ID, MT, NM, NV, OR, UT, WA, WY). Note: Percentages in the impact and financial hardships areas refer to the percentage of regional respondents to that time frame's survey (e.g., 3 of 6 Midwest libraries said COVID had a major impact in August 2020, which is 50%). Percentages in the Libraries responding area are percentages of the total number of respondents to each time frame's survey.

**Table 3:** Sentiment analysis of free-text responses to each survey. Both the number of uniquely used words and the total number of words used with positive and negative sentiments are captured.

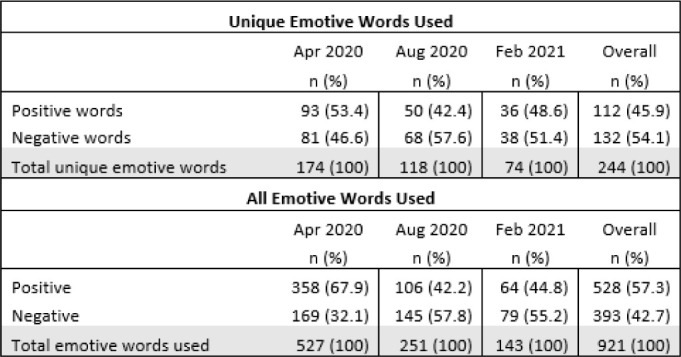

**Table 4:** Ten areas analyzed using staff as a lens for understanding the connection between codes.

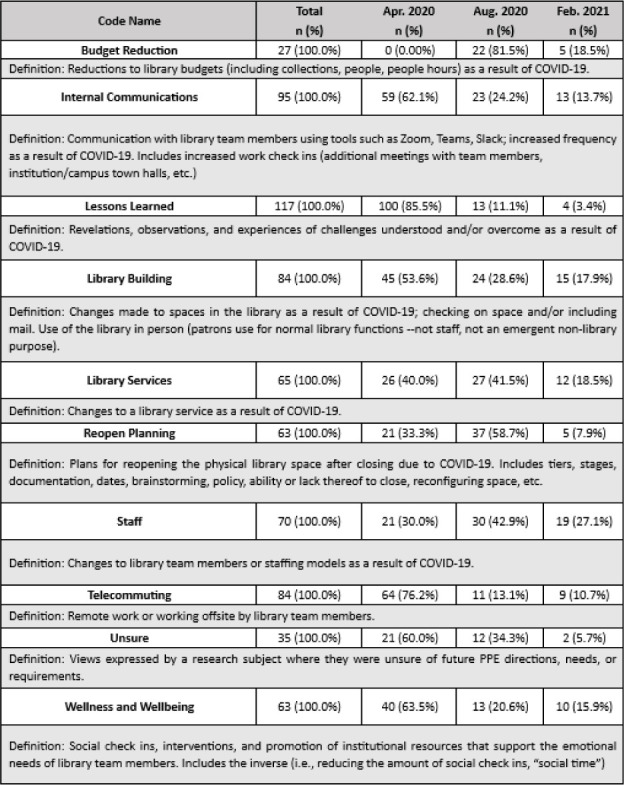

An additional post-hoc sentiment analysis ascertained the frequency of positive (e.g., amazing, fantastic) and negative (e.g., disheartening, frustrating) words in each data set. To create the data set, free text responses for each survey were copied into a Microsoft Word document and converted to a list for each word. Using Excel, the words were alphabetized and subtotaled to provide the overall number of times each appeared in each corpus. Words were individually reviewed for positive, negative, or neutral emotive content. Words with the same stem were conflated into one entry (e.g., encourag* for encouraged, encouraging, encouragement). For each period, totals were calculated to determine percentages of positive and negative words, both overall and unique words.

## RESULTS

Of the 193 possible AAHSL libraries represented and able to respond to the April 2020 survey, 45 (23.3%) responded to the first survey. Of those, 37 respondents answered at least one non-demographic question. Libraries represented 23 states and the District of Columbia; no responses were received from Canada. Twenty-six of the 37 respondents (70.3%) answered the August 2020 follow-up survey, and 16 answered the February 2021 survey. A total of 1,153 codes were applied to the open text from the 3 surveys (Table 3).

In April 2020, 21 (56.8%) of responding libraries' physical spaces were completely closed. An additional 13 (35.1%) libraries' physical spaces were closed to their constituencies, but one or more staff were working in the building (see Table 1). Only 1 (2.7%) library remained open with limited staffing in April 2020. Though more libraries in the East and Midwest were fully closed, in the South and West, the majority were physically closed to patrons but had at least one staff person working (62.5% and 55.6%, respectively). With the exception of the 1 library that remained open and staffed, libraries closed between March 12 and April 6, the majority (n=21, 56.8%) closing the week of March 15–21, 2020 (Table 1).

Overall, libraries found transitioning to a remote environment easy or very easy (n=24, 64.9%), with some types of work substantially easier to transition than others (see Figure 1a).

**Figure 1a:**
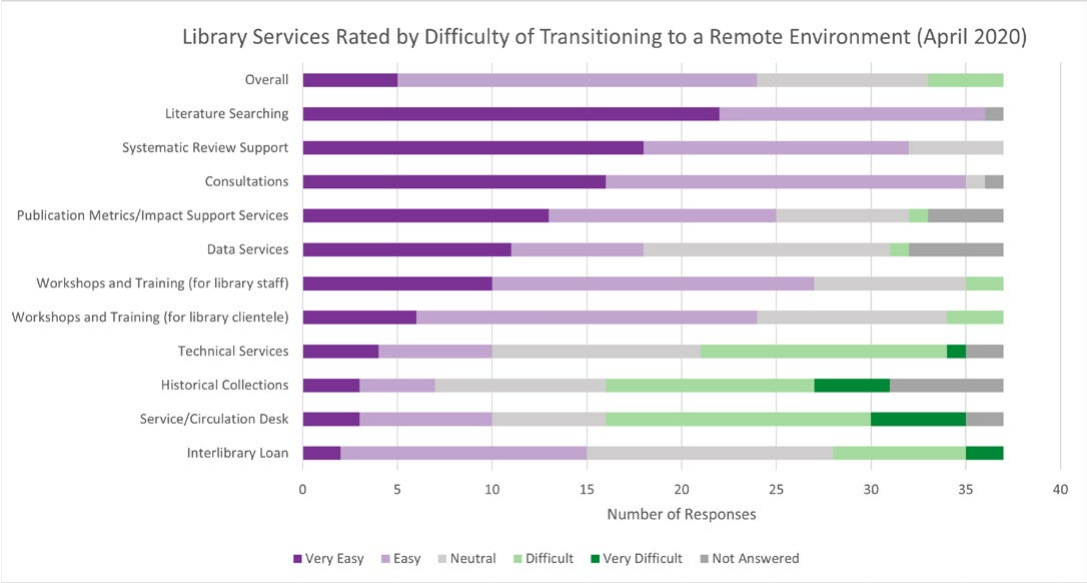
Library Services rated by difficulty of transitioning to a remote environment in April 2020.

In particular, literature searching, consultations, and systematic review services were considered the easiest to move online, whereas transitioning the circulation desk and its attendant services, historical collections, technical services, and interlibrary loan were considered the most difficult. More than half of respondents (n=19, 54.3%) found transitioning the service or circulation desk to be difficult or very difficult (see Figure 1b).

**Figure 1b:**
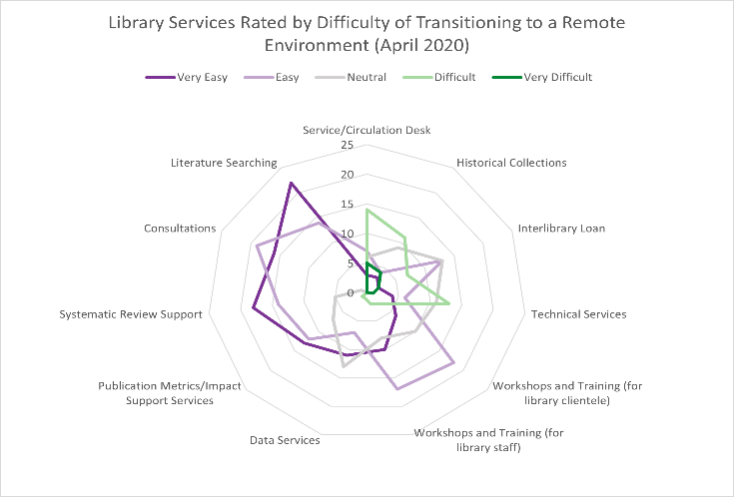
Radar chart of library services rated by difficulty of transitioning to a remote environment in April 2020. The radar chart does not include data for non-responses to highlight better the levels of difficulty for each service type.

A slightly higher percentage of libraries thought transitioning workshops and trainings to online formats was easier for those designed for library staff (n=27, 73.0%) than those created for library clientele (n=24, 64.9%).

By August 2020, many libraries were either open or had a planned opening date (n=13, 50.0%). A few libraries reopened or partially reopened their 24/7 spaces, and one library was fully open to the public without restriction. Two were open to members of the public by appointment. For those libraries that had reopened in some form, six noted they were operating with reduced hours, and 15 libraries, including some that did not reopen, had limited staff in the building (see Table 2). Fourteen libraries (53.8%) believed COVID-19 had a major impact on their operations the previous month; only one library did not see an impact in that time frame (see Table 2). Libraries commonly faced financial impacts from the pandemic, such as hiring freezes on new positions (n=17, 65.4%), reductions in collections (n=13, 50%), and having to rescind posted positions (n=10, 38.5%).

In February 2021, nearly all responding libraries were open or had a known opening date (n=13; 81.3%). Libraries continued to operate with limited staffing, reduced hours, and restrictions on access to members of the public (Table 2). Only four (25.0%) libraries believed the COVID-19 pandemic had a major impact on their library in the past month, versus ten (62.5%) that had seen moderate or minor impact (see Table 2). Similar to August 2020, only one library saw no impact from the pandemic in the prior month. By February 2021, more financial impacts on libraries were known. More libraries faced furloughs (n=5, 31.3%) and benefits reductions (n=2, 12.5%) than in August 2020.

The post-hoc sentiment analysis (Table 3) revealed a pattern of early pandemic positivity. Both the number of unique positive words and the total number of positive words were higher than negative words (53.4% of unique emotive words were positive; 67.9% of all emotive words used were positive). The early pattern of pandemic positivity is illustrated by the variance of positive and negative of all emotive words, which accounted for a 36% differential in April 2020. This pattern reversed by August 2020, where negative words were more prevalent (57.6% of unique emotive words were negative; 57.8% of all emotive words were negative) and only slightly tempered by February 2021, by which the number of uniquely used positive and negative words were almost equivalent (36 positive versus 38 negative). The total number of times the words were used, however, remained skewed (55.2% of all emotive words were negative).

## QUALITATIVE ANALYSIS

The goal of the qualitative analysis was to identify themes that emerged from the data describing the evolution of health sciences libraries during the pandemic. Initial codes, including their definitions, were first developed based on expected themes derived from the survey questions. Code distribution in Dedoose assisted in identifying important themes from the data. Library staff was identified as an important theme among all codes and used as a lens for understanding the connection between other codes in the data. Table 4 provides a summary of the code distribution and how they relate to remote library services, library facilities, and planning to reopen; internal communication and wellness and well-being; lessons learned, skills needed, and telecommuting; budget reduction and uncertainty; and issues related to equity.

### Remote library services, library facilities, and planning to reopen

By April 2020, most libraries had moved to remote and hybrid service models as universities shut down in response to growing concerns due to the pandemic. Libraries described the transition to remote work environments as a smooth process, expressing pride in staff response to the pandemic. Adjectives frequently used include “agile,” “quick to adapt,” and “resourcefulness.” According to one librarian, “I have been so proud of the staff at the health sciences library—they have pitched in wherever they are needed and have adapted to providing service in new ways… And we've also learned that we like each other and miss seeing each other in real life!”

Online conferencing tools greatly enabled the transition to remote service models. As libraries pivoted to new service models, common challenges included staff not having the current computing equipment at home and not all positions being well suited for remote work environments. Some respondents noted their emergency planning did not fully prepare them for this transition or did not include a “pandemic.” Multiple respondents expressed some sense of how this transition might have long-term impacts on how library work was conducted. Further, there was recognition some library jobs in the future may always have some or fully remote statuses.

When it came to library facilities, libraries described developing processes for some basic functions that could not be done remotely, including checking the mail and general facility checks. Even in the early stages of the pandemic, the connection between libraries, space, and safety was evident. One librarian stated, “I think we miss the space, and, at the same time, we are a bit anxious about how it will work to return to that space.” Protection was a consistent theme and considered needs related to masking, physical distancing, and other safety guidelines. Many discussed what the future space might look like, considering revised service hours, staggered staffing models, or the needs for reconfiguring to accommodate pandemic requirements. In some cases, library space was converted to support needs of the pandemic. Examples include providing space for a COVID-19 command center, nursing station patient area, and study locations for students without adequate internet access.

In August 2020, it was clear remote or hybrid services models would continue for an undetermined period for some. The state of library openness and staffing varied greatly. Some libraries had begun to open 24-hour space in some capacity but were conducting most core services remotely. Libraries with on-site services at this time did so with caution and reduced staffing and hours. One library shared, “We've removed many of the tables and chairs in the library to allow for social distancing, and our group study rooms are now by reservation only, with most of them only allowing one person at at (*sic*) time to use them.” Desire for more support and information from the institution continued to be a theme, with one library stating, “At this time, we're waiting on additional funding for security, cleaning, and student employees to be able to set our hours.”

Regardless of service model, libraries felt providing core services remotely had become routine. However, as services continued to evolve, the logistics of managing all operations for an extended period during a pandemic was creating new challenges, such as the need to conduct administrative business primarily virtually or managing on-site needs, often with staggered staffing models. Many libraries developed unique on-site services such as curbside pickup or in-person services by appointment. Some libraries continued to be closed to patrons. Those closed often had staff come in to manage services that could not be managed in a remote environment. Additionally, there was a desire for more communication and support from the institution.

Individually, libraries continued to evolve throughout the pandemic; however, no one best model emerged from the data. It is clear libraries were evolving based on local organizational requirements, which varied due to institutional policies, reporting structure, and local needs. By February 2021, most libraries expressed having some on-site services, albeit with core services usually provided in a hybrid environment. Instead, most libraries appeared to focus on the fall of 2021, when many institutions were planning some level of in-person instruction. At this time, vaccines were made available to certain populations, and there was great hope for some return to normalcy in the near future.

### Internal communication and wellness and well-being

In April 2020, many libraries reported increasing the frequency and types of internal communication, usually in the form of increased informal check-ins, more meetings (due to lack of physical interaction), and of course, more email communications. The use of technology to facilitate these increased communications—such as Zoom, Teams, Slack, and FaceTime—was mentioned for one-on-one meetings, group meetings, and broader town halls to share internal communications. Some respondents noted they used multiple modalities for communicating with staff based on staff preferences and/or the type of message conveyed. Along with increased “work” communication among folks in the libraries, informal options (happy hours, open hours) increased to help staff stay in touch. Some libraries also conducted staff development exercises and retreats. Direct communication increased “so there is no debating when we say something,” as one librarian put it, as well as debriefs after central institutional messages informed library staff specifically how new policies would affect the library.

Aside from these internal communications, many talked about wellness programming at their institution, either built upon existing programs or with recent investments in emotional support resources. Many libraries instituted online activities in which all employees could participate and connect, such as Zoom birthday celebrations, chair yoga, guided meditation, coffee/tea breaks, virtual lunches, variety hour, a friend's goat via Zoom, and virtual happy hours. Some adjusted or shortened meeting times to start fifteen minutes after the hour, and one respondent mentioned awarding administrative leave to employees. A few respondents focused on modeling wellness for their employees: deliberately encouraging it as part of the workday, targeting conversations so staff is asked how they are doing (not what they are accomplishing), and instituting, for example, “weekly reminders that expectations are being reset so people don't need to be extra-productive.”

By August 2020, responses to the survey demonstrated “internal communications” and “wellness and well-being” were mentioned much more frequently together than in April. While some libraries continued hosting virtual trivia events and informal meetings, engagement leveled off for other libraries. Using online technology tools for informal communication was still a necessity but increases to communication remained the same or slowed down. Topics of internal communication began to shift a bit, with libraries starting to talk more about the library budget and reopening plans. At least one library started conversations around “deeper issues related to COVID, systemic racism, anti-racism work, and equity as [the] physical return to workplace bec[ame] more likely for library assistants/staff but not for librarians.”

Later, in February 2021, some libraries did less frequent check-ins and, when commenting on additional engagement opportunities, mentioned, “there was not much enthusiasm for carving out more time.” Technology tools were an assumed necessity; however, people were getting burned out on Zoom meetings and realized a need to reduce meetings while still keeping people in touch and engaged. This response sums up the tenor of libraries at this point well: “The longer this pandemic and remote work situation continues, the more difficult it is to keep up morale and healthy/good communication dynamics. Skills that colleagues and managers developed do not automatically translate from in-person to remote environments.”

### Lessons learned, skills needed, and telecommuting

In April 2020, overwhelmingly, library administrative leaders gushed about how “fantastic,” “thoughtful,” “engaged,” and service-focused their staff is. They noted staff adaptability, agility, and flexibility as new challenges arose at a very fast pace. One librarian noted, “It has been incredibly inspiring to work together.” The concepts of flexibility, adaptability, and resiliency came up repeatedly—these skills were also recognized by those outside the library. For example, one librarian commented, “We were very flexible, and our ability to help has highlighted our usefulness to the institution.” Another respondent noted, “My boss […] has been with the university for over 20 years [and …] he said he had never seen the library as engaged as they have been during these past few months as they were. It is evident to him that we are deeply embedded in the organization by how much we were asked to do.” One library found that at their traditional institution, there may now be a way forward to continue working remotely in some capacity. They also noted the pandemic might enable their library to “take a big jump ahead in terms of letting go of some legacy activities and adopting more forward-thinking services.”

One of the biggest lessons learned was much of the work academic health sciences library staff does can be done remotely. It was clear from responses that most can be productive teleworkers. Generally, the transition to telecommuting/working remotely wasn't a huge barrier to overcome. However, additional lessons learned centered around a need for better assessment of staff needs for working remotely—better equipped desks and chairs in their homes, other equipment/technology needs, and having better plans for working from home for an extended amount of time (including updating policies). Challenges emerged around job responsibilities that cannot be done remotely, a need for more cross-training amongst staff, and missing interpersonal connections and serendipitous conversations.

By August 2020, libraries' lessons learned were a bit more pragmatic and less optimistic. A major concern centered around space, both how to alter it for reopening and what changes meant for the future of maintaining library facilities. While some respondents noted working remotely may continue for the near future, concerns around employees' mental well-being, lack of team cohesiveness, and disconnection from one another were evident. Additionally, many expressed anxiety about returning to work, particularly the tension between some employees feeling vulnerable with others wanting to return as soon as possible. Libraries started to see a shift in more employees coming back into the building, albeit in various hybrid configurations.

By the third survey in February 2021, there was both fatigue (e.g., “It [the pandemic] is getting tiring”) and resignation about the new normal noticeable in responses. In a slightly more positive spin, one respondent noted focusing on quantifying and qualifying their library's worth with new metrics to demonstrate their value and productivity. While many mentioned they are functioning with hybrid staffing models, one respondent observed their librarians wanted to assess the use of the space to support and/or justify permanent remote work for public service librarians. Overall, library buildings were open, but many only with reduced seating (physically distanced), staggered staffing or rotating schedules, and adjusted or reduced open hours.

### Budget reduction and uncertainty

In April 2020, libraries already anticipated budget reductions due to the pandemic, but widespread reductions were not yet evident. For some libraries, there were no layoffs, cuts, furloughs, or hiring freezes at this point. By August 2020, that all changed. Nearly every library either experienced budget reductions or was anticipating them. As one library noted, “Increasing demands; decreasing resources. Sigh.” Budget cuts of 5–20% were already in place for some libraries, which struggled to find places to cut mid-year.

The most common challenge libraries faced was reductions to staffing, generally by eliminating open positions. Some libraries voluntarily did so as a way to deal with severe budget reductions and avoid layoffs, though institutions mandated the elimination of open positions in other cases. Libraries began to see the effects of early retirement incentives offered by their institutions that resulted in permanent workforce reduction. Most libraries were experiencing hiring freezes, with very few noting a process for exceptions to the freezes. Some libraries experienced furloughs for some or all staff, generally partial furloughs that required a certain number of days per year. Libraries with major staffing reductions began reducing services and hours. Other libraries with cuts began to renegotiate vendor contracts, eliminate discretionary spending, and stop purchasing books.

One of the major concerns for libraries in August 2020 was an aura of uncertainty. As one librarian reported, “We know cuts and cut-backs are coming, but we do not yet know what those might be and how deep they may be…. So much is still to be revealed.” For some libraries, there was an anticipated budget reduction for the 2020/21 fiscal year, with future additional reductions looming. Many libraries were asked to plan for major reductions, though it was unclear whether the plans would need to be implemented. Libraries needed to engage in scenario planning to anticipate different budget scenarios.

Libraries also faced uncertainty about the future of collections and staffing, with special concern for being able to appropriately staff library buildings once libraries began to reopen. For many libraries, there remained tremendous uncertainty about when reopening buildings would be possible or allowed by their institutions, requiring additional scenario planning to be able to move quickly if reopening became an option. As one respondent noted, “We are working on a very detailed, three-phased approach…. No date has been set. However, we are doing all the planning necessary to drop our plan into place whenever the university deems it safe to do so.”

By February 2021, institutions began to recover slightly, with many now allowing exceptions to hiring freezes through special processes and additional scrutiny. Previously reduced salaries were restored for some, though raises were still not in sight. Many libraries still had not recovered their staff, however, and continued to face furloughs, reduced workforces, and, in some cases, layoffs. A few libraries experienced a reduced collection budget. There was less overall uncertainty, as many libraries' budget cuts manifested (or did not), and most libraries' physical spaces reopened to some degree.

### Issues related to equity

As libraries shifted processes and operations from online to in-person, they consciously made decisions regarding equity—acknowledging disparities in financial circumstances, transportation options, health, dependents, concern for family members, job responsibilities, and parity between types of jobs as potential factors. With most library employees still working remotely in April 2020, some libraries continued to have staff in the building to help with interlibrary loans, digitization, or other tasks requiring employees to be physically present. Their presence of staff in physical spaces reflected the perceived difficulty many libraries felt in transitioning to some services remotely (such as circulation, interlibrary loan, and technical services), whereas many librarian services (reference, teaching, etc.) transitioned more easily.

In May 2020, George Floyd was murdered by police officers, setting off one of the United States' great reckonings with systemic racism. As Americans grappled with how to change systems for the improvement of all, library staff saw a need to increase efforts on equity, antiracism, inclusion, and justice and wrestled challenging questions about library work while remaining remote and at a distance from one another. One librarian commented: “Beyond the personal emotional toll of quarantine on library workers, the political-social events of the last few months have added on to already existing deep feelings of need for systemic change in library work and the perceived value of the MLIS…. It is very hard for individuals to feel so powerless and so passionate and so isolated.” As libraries spent more time discussing systemic racism, antiracism, and equity in the context of libraries and COVID-19, it was noticeable that “physical return to [the] workplace becomes more likely for library assistants/staff but not for librarians.”

As libraries began to open by August 2020, many libraries faced both reduced staffing levels and continuing interest in remote work for many—along with concerns about equity. Most libraries created rotations to allow people to work on-site some days and remotely on others. Some libraries specifically noted that there were separate rotations for staff, librarians, and administrators to ensure all had some on-site time. Others used more nuanced rotations, prioritizing those whose job responsibilities required physical work in the library and asking others—whose work duties could be accomplished remotely—to remain remote. These plans often meant librarians remained remote for longer or more frequently than other types of library employee, particularly public services librarians. Libraries also based schedules and work locations on other factors, such as inability to work from home due to financial circumstances or medical concerns, as reasons for encouraging remote work. Some libraries required the same number of days on site per week for all employees, and others had flexible policies to allow for varying responsibilities and circumstances. Libraries experiencing salary cuts or furloughs consistently applied deeper cuts and a greater number of furlough days to higher level or higher salaried positions for equity. By February 2021, most libraries had opened to some degree. Rotations continued for many, some of which continued prioritizing shared responsibilities for physical presence in libraries between librarians and staff, and others made determinations on staffing based on job responsibilities. For example, clinical librarians began returning to physical spaces to attend case conferences. Other libraries began seeing public services librarians' interest in permanent remote work grow, acknowledging the success of remote services as justification.

## DISCUSSION

“Except for my commute, everything is the same,” cut and pasted several times by one study participant, is emblematic of the state of academic health sciences libraries in the early stages of the pandemic. Almost synchronously, libraries made an enormous evolutionary pivot while attempting to maintain the same standards of service from virtual locations. Survey answers reflected this enormous change throughout the study as respondents described American academic health sciences libraries in a continuous state of change throughout the COVID-19 pandemic.

Like many organizations, emergency planning had not prepared libraries for the rapid response needed for a global pandemic or the transition to remote service environments. Since the pandemic is not a singular event, uncertainty became a consistent theme for libraries, which were forced to respond to an evolving set of organizational needs. Despite these challenges, data reveals how libraries quickly evolved as a cohort, often displaying similar characteristics with peer academic health sciences libraries. For example, almost all libraries had closed their doors by April 2020 and reopened at some capacity by February 2021. Early on, the themes were service transition, telecommuting, conferencing technology, and wellness and well-being. As these themes became norms, new themes emerged around budget concerns and planning for reopening. Significant heterogeneity also exists as the data demonstrates how libraries developed local solutions to complex problems. These factors, often influenced by institutional need, include access to print collections, space provisioning, and document delivery.

Libraries demonstrated significant flexibility and innovation, but not without concerns. Services conducted by librarians, such as literature searching and consultations, were better adapted to virtual environments, while services provided by paraprofessionals, such as technical services and interlibrary loans, were less suited for the transition. This disparity in service transition is partially a function of some services' connection to the physical space but also reflects an equitability gap in libraries. The least paid staff members were less likely to have work laptops or stable high-speed internet at home, thus more likely to be required to work onsite during the greatest time of uncertainty during the pandemic.

Throughout all three surveys, there was broad recognition the pandemic would forever alter academic health sciences libraries. Post-hoc sentiment analysis suggests libraries were initially positive during the transition in April, felt more despair due to uncertainty in August, and then began to rebound in February 2021—likely due to the stabilization of new library service models, the emergence of vaccines, and plans to return to campus. The pandemic has continued to evolve and reshape global society ever since. These evolutions are likely to impact libraries for the foreseeable future, and the unknown is creating more questions than answers. For example, will there be another disruptive variant, and/or when will the pandemic evolve into an endemic virus? How will library staff and patrons continue to be impacted by the pandemic? The design and delivery of library services will most likely reflect changes in how patrons expect to use library services and resources. Within libraries, some changes may occur due to societal changes. For example, will a workforce shortage increase the demand for flexible work schedules and as a result, will some library positions become fully remote? The pendulum for norms within health sciences libraires may swing several times prior to stabilizing in a post-pandemic world.

The longitudinal, multisite nature of this study and the focus on using library staff as a lens for interpretation is unique. Other literature on the impact of COVID-19 on North American academic health sciences libraries has generally focused on case studies of libraries [6–13], descriptions of particular COVID-19-specific resources or services developed [14–17], or captured data at one time [18, 19]. Similar themes, however, emerged from this study and from others, which may point to the robustness of the results. For example, all the case studies discussed libraries where closures and moves to remote work occurred in March [6–12]. Those that returned to the building in 2020 opened after intense planning, generally with limited, staggered staffing and shorter hours [7, 9–12]. Nearly all noted transitioning reference services and teaching online was relatively easy, and some noted their reference staff found working from home preferable due to the ability to accomplish more [6–12]—commenting specifically on the use of technology like Slack and Teams or regular check-ins via video conferencing as ways to increase internal communication and staff well-being.

A major difference, however, is case studies tended to have less discussion of managerial considerations and equity concerns. There was additional focus on positivity and successes, even for those articles that spanned the August 2020 time frame where negativity and uncertainty seemed to peak in this analysis.

## LIMITATIONS

Only health sciences libraries in the United States responded to the invitation to participate in the study. Diminished participation between the three surveys created potential gaps in understanding the full impact of the pandemic on academic health sciences libraries. The quantitative survey instrument administered varied slightly between the first survey (April 2020) and surveys two (August 2020) and three (February 2021). This planned survey distribution allowed for themes to emerge over time and the inclusion of relevant questions, but this approach limits direct comparison between the three instruments. It is possible the interpretation of terms might differ between respondents and researchers, inadvertently creating bias in the responses collected. Data collection, analysis, and interpretation of findings relied on the investigators of this study.

## CONCLUSIONS

In March 2020, as many universities limited onsite access, health sciences libraries closed their doors to accommodate social distancing. Innovations by libraries during the early stages of the pandemic are having a long-term impact on library culture and the delivery of services. Even as libraries returned to in-person services, elements of telecommuting, using online conferencing software, safety precautions, and monitoring of staff well-being persisted. At the time of this writing, it is clear the pandemic will be with us for some time to come and continue creating substantial impacts on society and library services. This study ended data collection prior to the emergence of the Delta and Omicron variants. Further study is needed to determine the full extent of the pandemic on health sciences libraries. What is clear from the data is health sciences libraries demonstrated an enormous amount of resilience throughout the pandemic and the evolution between April 2020 and February 2021 will have a lasting impact for years to come. Insights gained from this study will assist libraries in preparing for future significant events and inform research, including the delivery of library services and equity among staff.

## Data Availability

Data associated with this article are available in the supplemental materials and at IUPUI ScholarWorks (https://scholarworks.iupui.edu/handle/1805/27513). Permission to share individual responses from this study was not provided by our IRB; therefore, that information cannot be made available at this time.

## References

[1] Adedoyin OB, Soykan E. Covid-19 pandemic and online learning: the challenges and opportunities. Interact Learn Environ [Internet]. 2020 [cited 2021 Dec 22]. https://www.tandfonline.com/doi/abs/10.1080/10494820.2020.1813180.

[2] Cucinotta D, Vanelli M. WHO Declares COVID-19 a Pandemic. Acta Bio Medica Atenei Parm [Internet]. 2020 [cited 2021 Dec 22];91(1):157. /labs/pmc/articles/PMC7569573/. 10.23750/abm.v91i1.9397 PMC7569573 32191675

[3] Austin CP, Jonson S, Kurilla MG. Foreword to the JCTS COVID-19 special issue. J Clin Transl Sci [Internet]. 2021 [cited 2021 Dec 9];5(1). https://www.cambridge.org/core/journals/journal-of-clinical-and-translational-science/article/foreword-to-thejcts-covid19-special-issue/4B9DBEC84DA2CF0FD96910A37A3C8CF3. 10.1017/cts.2021.400 PMC8190713 34164155

[4] Kim CS, Lynch JB, Cohen S, Neme S, Staiger TO, Evans L, Pergam SA, Liu C, Bryson-Cahn C, Dellit TH. One Academic Health System's Early (and Ongoing) Experience Responding to COVID-19: Recommendations From the Initial Epicenter of the Pandemic in the United States. Acad Med [Internet]. 2020 Aug 1 [cited 2021 Dec 22];95(8):1146–8. /labs/pmc/articles/PMC7176258/. 32282371 10.1097/ACM.0000000000003410 PMC7176258

[5] Covid-19 Pandemic Timeline Fast Facts. CNN [Internet]. [cited 2021 Dec 22]. https://www.cnn.com/2021/08/09/health/covid-19-pandemic-timeline-fast-facts/index.html.

[6] Weeks A, Houk KM, Nugent RL, Corn M, Lackey M. UNLV Health Sciences Library's Initial Response to the COVID-19 Pandemic: How a Versatile Environment, Online Technologies, and Liaison Expertise Prepared Library Faculty in Supporting Its User Communities. Med Ref Serv Q. 2020 Oct 1;39(4):344–58. 33085950 10.1080/02763869.2020.1826197

[7] Gotschall T, Gillum S, Herring P, Lambert C, Collins R, Dexter N. When One Library Door Closes, Another Virtual One Opens: A Team Response to the Remote Library. Med Ref Serv Q. 2021;40(1):11–22. 33625335 10.1080/02763869.2021.1873612

[8] Garg S, Kim L, Whitaker M, O'Halloran A, Cummings C, Holstein R, Prill M, Chai SJ, Kirley PD, Alden NB, Kawasaki B, Yousey-Hindes K, Niccolai L, Anderson EJ, Openo KP, Weigel A, Monroe ML, Ryan P, Henderson J, Kim S, Como-Sabetti K, Lynfield R, Sosin D, Torres S, Muse A, Bennett NM, Billing L, Sutton M, West N, Schaffner W, Talbot HK, Aquino C, George A, Budd A, Brammer L, Langley G, Hall AJ, Fry A. Hospitalization Rates and Characteristics of Patients Hospitalized with Laboratory-Confirmed Coronavirus Disease 2019 — COVID-NET, 14 States, March 1–30, 2020. MMWR Morb Mortal Wkly Rep [Internet]. 2020 Apr 17 [cited 2020 May 26];69(15):458–64. http://www.cdc.gov/mmwr/volumes/69/wr/mm6915e3.htm?s_cid=mm6915e3_w. 32298251 10.15585/mmwr.mm6915e3 PMC7755063

[9] Lindsay JM, Petersen D, Grabeel KL, Quesenberry AC, Pujol A, Earl M. Mind like Water: Flexibly Adapting to Serve Patrons in the Era of COVID-19. Med Ref Serv Q. 2021;40(1):56–66. 33625333 10.1080/02763869.2021.1873622

[10] Howes L, Ferrell L, Pettys G, Roloff A. Adapting to Remote Library Services during COVID-19. Med Ref Serv Q. 2021;40(1):35–47. 33625328 10.1080/02763869.2021.1873616

[11] Mazure ES, Colburn JL, Wallace E, Justice EM, Shaw S, Stigleman S. Librarian Contributions to a Regional Response in the COVID-19 Pandemic in Western North Carolina. Med Ref Serv Q. 2021;40(1):79–89. 33625326 10.1080/02763869.2021.1873626

[12] Koos JA, Scheinfeld L, Larson C. Pandemic-Proofing Your Library: Disaster Response and Lessons Learned from COVID-19. Med Ref Serv Q. 2021;40(1):67–78. 33625324 10.1080/02763869.2021.1873624

[13] Cote MP, Donne EM, Hoover BD, Thormodson K. Teaching Instructional Technological Change to Medical School Faculty: A COVID-19 Case Study. Med Ref Serv Q. 2020 Oct 1;39(4):406–10. 33085947 10.1080/02763869.2020.1826234

[14] Haugh D. Communicating with medical library users during COVID-19. J Med Libr Assoc. 2021;109(1):107–11. 33424471 10.5195/jmla.2021.1003 PMC7772968

[15] Callaway J. The Librarian Reserve Corps: An Emergency Response. Med Ref Serv Q. 2021;40(1):90–102. 33625329 10.1080/02763869.2021.1873627

[16] Sullo E, Brody S. Providing Information to Support COVID-19 Pandemic Response: Academic Medical Librarians' Roles in Creating an Intelligence Report. Med Ref Serv Q. 2021;40(1):23–34. 33625323 10.1080/02763869.2021.1873613

[17] Charney RL, Spencer A, Tao D. A Novel Partnership Between Physicians and Medical Librarians During the COVID-19 Pandemic. Med Ref Serv Q. 2021;40(1):48–55. 33625330 10.1080/02763869.2021.1873617

[18] Clifton VL, Flathers KM, Brigham TJ. COVID-19–Background and Health Sciences Library Response during the First Months of the Pandemic. Med Ref Serv Q. 2021;40(1):1–10. 33625334 10.1080/02763869.2021.1873611

[19] Charbonneau DH, E. V. Effects of the COVID-19 Pandemic on Academic Medical Library Services. In: Paper presented at: MLA ’21 vConference; 2021 May 24–27 Virtual. 2021.

