# Supplementary material for: Except for my commute, everything is the same: the shared lived experience of health sciences libraries during the COVID-19 pandemic
Source: J Med Libr Assoc. 2022 Oct 1;110(4):419–28. doi: 10.5195/jmla.2022.1475 (PMC10124594; doi:10.5195/jmla.2022.1475)
Supplement: Supplementary file 1 — Appendix A: Figures 1a and 1b [file jmla-110-4-419-s01.pdf]

**Appendix A: Figures 1a + 1b**

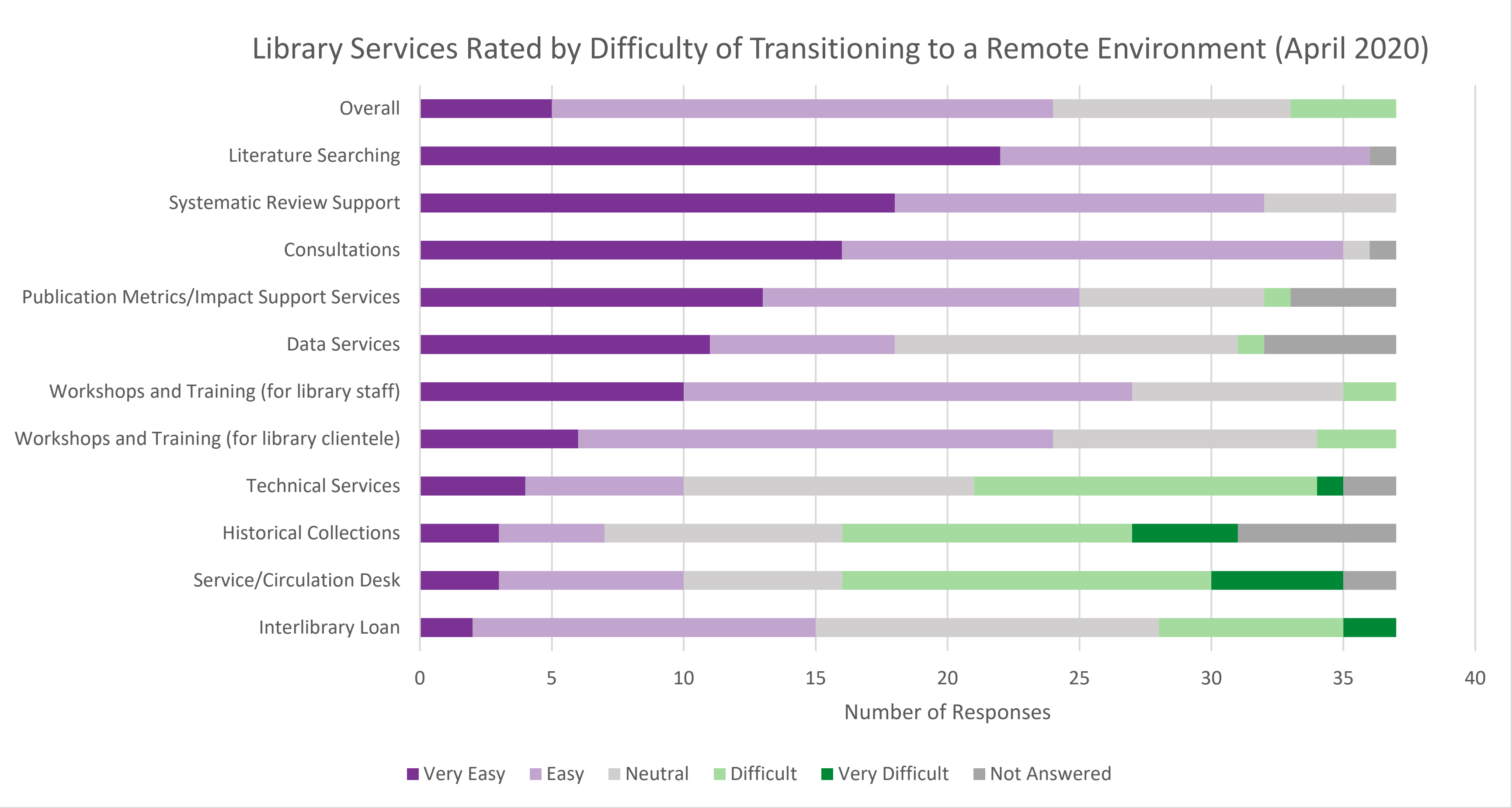

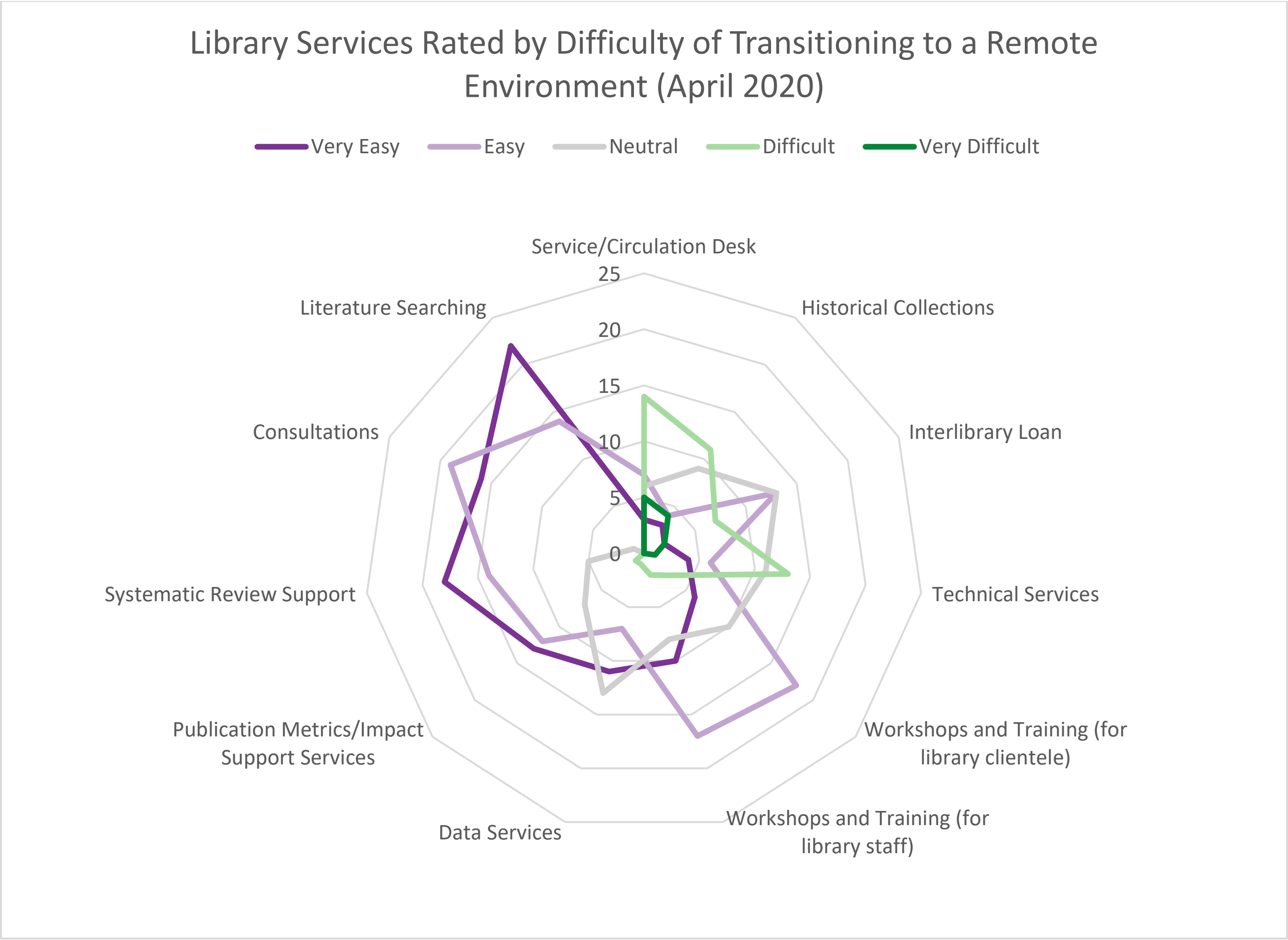


*This chart shows number of responses to the April 2020 survey.
