# Supplementary material for: Except for my commute, everything is the same: the shared lived experience of health sciences libraries during the COVID-19 pandemic
Source: J Med Libr Assoc. 2022 Oct 1;110(4):419–28. doi: 10.5195/jmla.2022.1475 (PMC10124594; doi:10.5195/jmla.2022.1475)
Supplement: Supplementary file 2 — Appendix B: Tables [file jmla-110-4-419-s02.pdf]

Appendix B: Tables

Table 1. April 2020 Library Status by Region

	East n (%)	Midwest n (%)	South n (%)	West n (%)	Total n (%)
Library is completely closed	9 (75.0)	5 (62.5)	3 (37.5)	4 (44.4)	21 (56.8)
Library is closed, but a 24 hour space is open	3 (25.0)	0 (0.0)	1 (12.5)	2 (22.2)	6 (16.2)
Library is closed, but one or more staff are regularly working in the facility	0 (0.0)	3 (37.5)	5 (62.5)	5 (55.6)	13 (35.1)
Library is open with reduced staffing	0 (0.0)	0 (0.0)	0 (0.0)	1 (11.1)	1 (2.7)
Total Libraries responding	12 (32.4)	8 (21.6)	8 (21.6)	9 (24.3)	37 (100.0)

Table 2. Impact of COVID-19 on library operations, finances, and staffing by region, comparing August 2020 and February 2021 responses.

	**East**		**Midwest**		**South**		**West**		**Total**	
	Aug 2020 n (%)	Feb 2021 n (%)	Aug 2020 n (%)	Feb 2021 n (%)	Aug 2020 n (%)	Feb 2021 n (%)	Aug 2020 n (%)	Feb 2021 n (%)	Aug 2020 n (%)	Feb 2021 n (%)
Libraries responding	7 (26.9)	3 (18.8)	6 (23.1)	3 (18.8)	6 (23.1)	6 (37.5)	7 (26.9)	4 (25.0)	26 (100)	16 (100)
In the past month, how much as COVID-19 affected the operations of your library?										
Major impact	4 (57.1)	1 (33.3)	3 (50.0)	1 (33.3)	4 (66.7)	0 (0.0)	3 (42.9)	2 (50.0)	14 (53.8)	4 (25.0)
Moderate impact	2 (28.6)	1 (33.3)	2 (33.3)	1 (33.3)	1 (16.7)	4 (66.7)	1 (14.3)	1 (25.0)	6 (23.1)	7 (43.8)
Minor impact	1 (14.3)	1 (33.3)	1 (16.7)	0 (0.0)	1 (16.7)	1 (16.7)	1 (14.3)	1 (25.0)	4 (15.4)	3 (18.8)
Neutral	0 (0.0)	0 (0.0)	0 (0.0)	1 (33.3)	0 (0.0)	0 (0.0)	0 (0.0)	0 (0.0)	0 (0.0)	1 (6.3)
No impact	0 (0.0)	0 (0.0)	0 (0.0)	0 (0.0)	0 (0.0)	1 (16.7)	1 (14.3)	0 (0.0)	1 (3.8)	1 (6.3)
Which of the following financial hardships are applicable for your library?										
Furloughs	1 (14.3)	1 (33.3)	2 (33.3)	1 (33.3)	0 (0.0)	1 (16.7)	1 (14.3)	2 (50.0)	4 (15.4)	5 (31.3)
Layoffs	2 (28.6)	3 (100.0)	1 (16.7)	0 (0.0)	0 (0.0)	0 (0.0)	0 (0.0)	0 (0.0)	3 (11.5)	3 (18.8)
Hiring freeze (new positions)	6 (85.7)	2 (66.7)	3 (23.1)	1 (33.3)	3 (23.1)	4 (66.7)	5 (71.4)	3 (75.0)	17 (65.4)	10 (62.5)
Hiring freeze (rescind posted positions)	4 (57.1)	1 (33.3)	0 (0.0)	0 (0.0)	3 (50.0)	0 (0.0)	3 (42.9)	1 (25.0)	10 (38.5)	2 (12.5)
Salary reductions	1 (14.3)	1 (33.3)	2 (33.3)	1 (33.3)	1 (16.7)	1 (16.7)	1 (14.3)	0 (0.0)	5 (19.2)	3 (18.8)
Benefits reductions	1 (14.3)	1 (33.3)	0 (0.0)	1 (33.3)	0 (0.0)	0 (0.0)	0 (0.0)	0 (0.0)	1 (3.8)	2 (12.5)
Collections reductions	2 (28.6)	3 (100.0)	5 (83.3)	2 (66.7)	2 (33.3)	2 (33.3)	4 (57.1)	0 (0.0)	13 (50.0)	7 (43.8)

Regions are as defined by the Association of Academic Health Sciences Libraries: East (CT, DC, DE, MA, MD, ME, NH, NJ, NY, PA, RI, VA, VT); South (AL, AR, FL, GA, LA, MS, NC, OK, PR, SC, TN, TX, VI, WV); Midwest (IA, IL, IN, KS, KY, MI, MN, MO, ND, NE, OH, SD, WI); West (AK, AZ, CA, CO, HI, ID, MT, NM, NV, OR, UT, WA, WY). Note: Percentages in the impact and financial hardships areas refer to the percentage of regional respondents to that time frame's survey (e.g., 3 of 6 Midwest libraries said COVID had a major impact in August 2020, which is 50%). Percentages in the Libraries responding area are percentages of the total number of respondents to each time frame's survey.

Table 3. Sentiment analysis of free-text responses to each survey. Both the number of uniquely used words and the total number of words used with positive and negative sentiments are captured.

Unique Emotive Words Used				
	Apr 2020<br>n (%)	Aug 2020<br>n (%)	Feb 2021<br>n (%)	Overall<br>n (%)
Positive words	93 (53.4)	50 (42.4)	36 (48.6)	112 (45.9)
Negative words	81 (46.6)	68 (57.6)	38 (51.4)	132 (54.1)
Total unique emotive words	174 (100)	118 (100)	74 (100)	244 (100)
**All Emotive Words Used**				
	Apr 2020<br>n (%)	Aug 2020<br>n (%)	Feb 2021<br>n (%)	Overall<br>n (%)
Positive	358 (67.9)	106 (42.2)	64 (44.8)	528 (57.3)
Negative	169 (32.1)	145 (57.8)	79 (55.2)	393 (42.7)
Total emotive words used	527 (100)	251 (100)	143 (100)	921 (100)

Table 4. Ten areas analyzed using staff as a lens for understanding the connection between codes.

Code Name	Total<br>n (%)	Apr. 2020<br>n (%)	Aug. 2020<br>n (%)	Feb. 2021<br>n (%)
**Budget Reduction**	27 (100.0%)	0 (0.00%)	22 (81.5%)	5 (18.5%)
Definition: Reductions to library budgets (including collections, people, people hours) as a result of COVID-19.				
**Internal Communications**	95 (100.0%)	59 (62.1%)	23 (24.2%)	13 (13.7%)
Definition: Communication with library team members using tools such as Zoom, Teams, Slack; increased frequency as a result of COVID-19. Includes increased work check ins (additional meetings with team members, institution/campus town halls, etc.)				
**Lessons Learned**	117 (100.0%)	100 (85.5%)	13 (11.1%)	4 (3.4%)
Definition: Revelations, observations, and experiences of challenges understood and/or overcome as a result of COVID-19.				
**Library Building**	84 (100.0%)	45 (53.6%)	24 (28.6%)	15 (17.9%)
Definition: Changes made to spaces in the library as a result of COVID-19; checking on space and/or including mail. Use of the library in person (patrons use for normal library functions --not staff, not an emergent non-library purpose).				
**Library Services**	65 (100.0%)	26 (40.0%)	27 (41.5%)	12 (18.5%)
Definition: Changes to a library service as a result of COVID-19.				
**Reopen Planning**	63 (100.0%)	21 (33.3%)	37 (58.7%)	5 (7.9%)
Definition: Plans for reopening the physical library space after closing due to COVID-19. Includes tiers, stages, documentation, dates, brainstorming, policy, ability or lack thereof to close, reconfiguring space, etc.				

**Staff**	70 (100.0%)	21 (30.0%)	30 (42.9%)	19 (27.1%)
Definition: Changes to library team members or staffing models as a result of COVID-19.				
**Telecommuting**	84 (100.0%)	64 (76.2%)	11 (13.1%)	9 (10.7%)
Definition: Remote work or working offsite by library team members.				
**Unsure**	35 (100.0%)	21 (60.0%)	12 (34.3%)	2 (5.7%)
Definition: Views expressed by a research subject where they were unsure of future PPE directions, needs, or requirements.				
**Wellness and Wellbeing**	63 (100.0%)	40 (63.5%)	13 (20.6%)	10 (15.9%)
Definition: Social check ins, interventions, and promotion of institutional resources that support the emotional needs of library team members. Includes the inverse (i.e., reducing the amount of social check ins, "social time")				
